# Can provision of near vision glasses as an early intervention improve visual outcomes in infants at risk of perinatal brain insult? The Babies in Glasses (BiG) randomised feasibility trial

**DOI:** 10.1136/bmjopen-2025-107894

**Published:** 2026-02-16

**Authors:** Raimonda Bullaj, Leigh Dyet, Subhabrata Mitra, Catey Bunce, Caroline S Clarke, Kathryn Saunders, Naomi Dale, Anna Horwood, Cathy Williams, Helen St Clair Tracy, Neil Marlow, Richard Bowman

**Affiliations:** 1 Ophthalmology, Great Ormond Street Hospital NHS Trust, London, UK; 2 Institute for Women’s Health, University College London Hospitals NHS Foundation Trust, London, UK; 3 University College London, London, UK; 4 London School of Hygiene & Tropical Medicine Faculty of Infectious and Tropical Diseases, London, UK; 5 Research Department of Primary Care and Population Health, University College London, UCL Medical School, UK; 6 Biomedical Sciences Research Institute, Ulster University, Coleraine, UK; 7 Great Ormond Street Hospital for Children, London, UK; 8 School of Psychology, University of Reading, Reading, UK; 9 Bristol Medical School, University of Bristol, Bristol, UK; 10 University of Bristol, Bristol, UK; 11 School of Medicine, University of St Andrews, St Andrews, UK; 12 International Centre for Eye Health, London School of Hygiene & Tropical Medicine, London, UK; 13 Institute of Child Health, UCL, London, UK

**Keywords:** Paediatric ophthalmology, NEONATOLOGY, Developmental neurology & neurodisability

## Abstract

**Objectives:**

We conducted a feasibility study to evaluate the feasibility of recruiting patients to examine the effect of near vision glasses in young infants at risk of cerebral visual impairment.

**Design:**

A three-arm, parallel-group, open-label randomised feasibility trial.

**Setting:**

Tertiary neonatal intensive care in London, UK.

**Participants:**

We included babies born before 29 weeks of gestation or at full term with hypoxic ischaemic encephalopathy. Babies who needed ongoing inpatient care, with established eye anomalies or with very high refractive errors at baseline (<−6.00 dioptres (D) or >+8.00D) were not included. Infants with retinopathy of prematurity were not excluded.

**Interventions:**

At 8 weeks corrected age, we allocated 18 infants to wear glasses (+3.00D over full cycloplegic refraction) immediately (intervention 1), 18 to wear the same glasses at 16 weeks (intervention 2) and 19 infants were allocated to standard treatment (no glasses).

**Outcomes:**

Recruitment and retention of study participants (primary), compliance wearing glasses, preferential-looking visual acuity (with glasses) and visual function as determined using A Test Battery of Child Development for Examining Functional Vision at 3-month and 6-month age post-term.

**Results:**

Of 70 eligible families, 55 consented and 34 attended baseline assessments, and 28 completed the study. Non-attendance was due mainly to prolonged inpatient stay, infant health and scheduling conflicts. Glasses were worn for similar periods in each group (Intervention 1: median 2 hours/day (95% CI 1 hour to 4 hours); Intervention 2: median 2 hours/day (95% CI 1.5 hours to 3 hours)). Visual acuity improved from baseline to 6 months. Mean (SE) LogMAR (Minimum Angle of Resolution) improvements were standard care: 0.47 (0.45); intervention 1: 0.66 (0.44); intervention 2: 0.37 (0.36). Among the 29 very preterm infants, there were similar findings: standard care: 0.35 (0.35); Intervention 1: 0.67 (0.47); Intervention 2: 0.34 (0.40). As a functional measure, object permanence was present at the following rates by randomised arm: standard care: 29%; whereas intervention 1: 56%; and intervention 2: 44% (OR intervention 1 vs standard care: 3.13 (95% CI 0.38 to 25.57), ie, not statistically significant).

**Conclusions:**

We demonstrate feasibility for a definitive RCT (randomized controlled trial) with good recruitment and retention and observed potential benefits for vision and development following the dispensing of glasses at 8 weeks post-term age compared with untreated controls. We identified methodological modifications to further improve recruitment processes for a future larger study.

**Trial registration numbers:**

ISRCTN14646770; NCT05048550.

**Strengths and limitations of this study**

- The study employed a rigorous randomised design, incorporating clearly defined eligibility criteria, standardised recruitment pathways, concealed allocation procedures and consistent protocols for delivering the intervention and assessing outcomes.
- Independent Data Monitoring and Ethics Committee oversight ensured case-by-case risk monitoring and ethical trial conduct.
- Use of clinically validated visual assessment tools enhances the reliability and clinical relevance of outcome measures.
- Extensive involvement of patient and public involvement throughout, including contributions influencing key changes and decisions necessary for progression to the next stages of research.
- A limitation of this study is that it was conducted at a single centre, which may limit the generalisability of the findings to other populations and clinical settings.

## Introduction

 Cerebral visual impairment (CVI) is defined as ‘a verifiable visual dysfunction which cannot be attributed to disorders of the anterior visual pathways or any potentially co-occurring ocular impairment’.^1^ In 2003, CVI accounted for half of the severe visual impairment cases among UK children,^2^ and half of all visual impairment in 2021.^3^ Even mild CVI can negatively impact educational outcomes and quality of life.^4^ CVI is particularly common after significant perinatal brain insults which affect around 5.14 children per 1000 live births,^5^ for example, those accompanying very preterm birth and hypoxic ischaemic encephalopathy (HIE) at term. Both conditions have a high risk for CVI and for long-term neurocognitive impairment that is detectable in infancy.^6^,^11^ There is a need for safe, simple and cost-effective interventions that reduce the prevalence or severity of CVI with potential to mitigate long-term disabilities.

Hubel identified the importance of a critical period in infancy for vision correction because of reduced neuroplasticity with advancing age.^12^ Although this seminal animal work was a model of deprivational amblyopia (involving prolonged eye closure), the understanding of the importance of early intervention has been applied successfully to other types of amblyopia such as strabismic and ametropic amblyopia. The latter refers to long-term visual impairment resulting from an early period of poorly focused vision due to uncorrected refractive error. As a result of this understanding, clinical guidelines have been developed for early provision of glasses for children with refractive errors that are regarded as clinically significant.^13^ However, there is no early screening programme for refractive error and in practice, glasses are typically prescribed at older ages for children with CVI to improve vision and educational outcomes at older ages. In contrast, surgery for cataract and squint is carried out early in infancy to avoid poor vision outcomes.^14^ It is recognised that young infants are often hypermetropic^15^ and have difficulty focusing consistently and accurately on near objects, such as the caregiver’s face or the infant’s hands—both important developmental stages. Typically developing infants quickly develop accurate accommodation (near focus ability), but emerging evidence suggests that children with a range of neurodevelopmental conditions, many of which are associated with CVI (such as Cerebral Palsy, Down syndrome and congenital Zika infection), experience significant difficulty with accommodation, and therefore optical blurring, even as they grow older.^9^,^1116 17^ These children benefit from the use of near vision glasses, especially in an educational setting.^16 18^ However, this poor accommodation early in visual development when near focus is important may produce long-term amblyopia. For instance, refractive amblyopia has been shown to impact pattern visual evoked potential responses.^19^ Poor accommodation in one eye due to Adie syndrome has been shown to cause amblyopia even in older, less vulnerable children,^20 21^ but we know that bilateral refractive errors can also cause bilateral ametropic amblyopia.

We hypothesised that early intervention with near-vision glasses provision, for infants at risk of CVI, may offer long-term benefits for vision and development. In this study, we determined the feasibility of early intervention using glasses for near-vision correction in infants at a high risk of CVI. For our experimental arm, we wanted to achieve the sharpest focus possible at near range (33 cm) and so we prescribed full cycloplegic refraction (including astigmatism) plus 3.00 dioptres (D) near addition as single vision near glasses. We wanted to compare this to standard clinical care, which is not to provide glasses at all at this age unless children are found to have refractive errors in excess of standardised thresholds.^13^

## Methods

We conducted a three-arm, parallel-group, open-label randomised feasibility trial (the Babies in Glasses study).

### Participants

We included families with infants born at less than 29 weeks of gestation and infants undergoing therapeutic hypothermia for HIE. Evidence of hypo-accommodation was not required. We excluded infants who remained inpatients at 8 weeks corrected age, those with congenital or developmental ocular abnormalities unrelated to the study (eg, genetic retinal diseases, cataracts requiring surgery or coloboma) or with very high refractive errors (spherical equivalent ≥−6.00D or +8.00D). Infants with retinopathy of prematurity (ROP) were not excluded.

### Design and setting

Recruitment, randomisation and research visits took place at University College London Hospital, London, UK. We recruited families in the Neonatal Intensive Care Unit (NICU), which cares for the full range of neonatal conditions. Visual assessments were conducted in the adjacent paediatric outpatient department.

### Patient and public involvement

A Patient and Public Involvement Committee was engaged throughout all stages of the study, contributing to the review of study materials, draft protocols, selection of outcomes, analysis and dissemination of results. Visual and neurodevelopmental assessments were initially planned for the same day; however, after consultation with our PPI group, these were held on separate days. The full details of the flow of participants through the study, patient and public involvement, sample size calculation and schedule of events are described in the study protocol*.*^22^

### Study enrolment

Families were screened on admission to NICU by research nurses and the study optometrist (RBu). After discussion, eligible families were contacted, the study was discussed and an information leaflet was given. RBu obtained written informed consent during the inpatient stay. Randomisation was performed by RBu approximately 1 week before the infant was 8 weeks post term age using an online service (‘Sealed Envelope’). Some subjects remained inpatients up until or beyond 8 weeks corrected age despite being scheduled for discharge at the point of randomisation and were therefore excluded. Subjects were allocated using a 1:1:1 ratio, stratified by clinical group (very preterm or HIE). Allocation was not disclosed to the parents before the baseline visit, ensuring any drop-out was not biased by allocation.

### Management of intervention

Allocation was to standard treatment (no glasses, unless high refractive error found, according to standard guidelines,^13^ myopia >−5.00, hypermetropia > +6.00, adjusted clinically in the presence of strabismus), near vision glasses (full cycloplegic refraction, including cylinder, +3.00 dioptres) at 8 weeks corrected age (intervention 1), or the same intervention at 16 weeks corrected age (intervention 2). Vision was evaluated at 20 weeks and 32 weeks corrected age (control and intervention 1) or at 28 weeks and 40 weeks (intervention 2), that is, 12 and 24 weeks post baseline assessment for all subjects.

Standardised vision outcome evaluations were conducted uncorrected and also corrected if glasses had been prescribed (intervention groups) and comprised preferential-looking visual acuity and A Test Battery of Child Development for Examining Functional Vision (ABCDEFV).^23^ Retinoscopy was used to evaluate both refraction and accommodation, with dynamic retinoscopy—using the Nott method^24^ —specifically applied for assessing accommodation. Vision and all ABCDEFV developmental assessments were assessed binocularly reflecting real-world functional vision use. In contrast, accommodation and refractive error were measured monocularly to ensure accurate, eye-specific data. The specialist paediatric optometrist further evaluated eye structure and functional vision, and parents completed a range of questionnaires.^22^

To encourage protocol adherence and ensure maximum benefit from the intervention, where glasses had been prescribed, parents/carers were advised that they should be worn for all waking hours, although we accepted that compliance would vary from one infant to another. Each parent/carer received a monthly telephone interview on varying days of the week, based on a semi-structured questionnaire. The interview addressed tolerability, compliance issues and provided further education on the use of glasses. The number of hours each infant was awake and the number of hours the glasses were worn were recorded.

### Outcome measures

The primary aim of this study was to assess the feasibility and acceptability of both (a) the intervention and (b) the trial methods.

### Primary outcome

The acceptability of recruitment to the randomised trial, defined by the proportion of eligible parents who consented to participate.

### Secondary outcomes

Feasibility

- Success rate of fitting and dispensing glasses with varying refractive corrections to infants aged 8 weeks vs 16 weeks age corrected for preterm birth as appropriate.
- Compliance with dispensed glasses wear for infants at 12 weeks and 24 weeks after intervention as appropriate.
- The proportion of families completing phone questionnaires on glasses compliance as a percentage of those in the intervention groups.
- Retention rate.

Clinical measures

- Visual acuity at 12 and 24 weeks compared with baseline acuity.
- Refractive outcomes at 12 and 24 weeks compared with baseline.
- Accommodative outcomes at 12 and 24 weeks compared with baseline.
- Evidence of mechanical trauma from prescribed glasses.
- ABCDEFV scores at baseline, 12 and 24 weeks postintervention.
- Determination of participant’s utilisation of healthcare resources using a modified Client Service Receipt Inventory (CSRI) form.

Infants allocated to standard care were evaluated alongside those receiving intervention 1. Observed differences in clinical outcomes between groups should be interpreted cautiously, as the study was not powered to detect statistically significant effects and no primary outcome was specified for hypothesis testing at this stage.

### Statistical analysis

The proportion of patients who accepted the offer of randomisation and the number of families who were lost to follow-up are reported with a 95% CI computed by the exact binomial method. Baseline characteristics are reported by treatment allocation using means and SD or medians and IQR as appropriate. Categorical data are reported as numbers and frequencies. We report the number of subjects missing data for any characteristic and did not impute. Participants were analysed according to the groups to which they were allocated, regardless of compliance, in line with an intention-to-treat approach. All harms observed during the study are reported by treatment arm. To examine for a signal of efficacy, we computed the difference in means for visual acuity between each treatment group and OR for components of ABCDEFV showing apparent differences between groups.

### Health economic analysis

The aim of this part of the feasibility study was to assess the feasibility of calculating the incremental cost per unit benefit of providing glasses compared with treatment as usual in the control arm in a future full RCT, from the perspective of the NHS (National Health Service) and Personal Social Services (PSS) as preferred by NICE (National Institute of Clinical Excellence). Time off work by the parents or other carers was also captured as it would be used in a future full trial to assess productivity losses. The objective was to determine and pilot appropriate resource-use data collection methods for a future full RCT. To this end, a targeted paediatric resource use questionnaire (RUQ) form was designed specifically for this population in collaboration with the rest of the study team, based on the CSRI. Descriptive statistics are reported here for resource use items and their levels of completion, by randomised group. In line with the analysis plan for this feasibility study, no formal comparisons of resource use across groups have been made.

The feasibility of obtaining data on the cost of the intervention itself is also reported as estimated costs of providing the intervention, by randomised group, first using raw unimputed costs and second where missing costs for frames and lenses have been imputed using mean values from the remaining participants.

### Oversight

The study was overseen by a Trial Steering Committee and an Independent Data Monitoring and Ethics Committee.

### Data collection

Data collection occurred through the use of a combination of structured clinical documentation, remote follow-up tools and administrative tracking systems to ensure comprehensive and coordinated data capture. Case report forms (CRFs) were completed by the researcher during in-person assessments to record clinical, health economic and developmental data as well as spectacle compliance and dispensing. In addition, administrative tracking sheets were used to coordinate and log all telephone contacts, including dates, call outcomes (eg, completed, rescheduled, no answer), and any key notes. Similarly, appointment booking logs were maintained to schedule, confirm and track attendance at in-person assessments, helping to monitor retention and visit completion rates. Reports of adverse events or harms were collected both during clinic visits and phone calls and were documented in designated safety sections of the CRFs. Information on the costs of measuring, prescribing, ordering and dispensing the glasses was captured to calculate the cost of providing the intervention. See online supplemental appendix for the RUQ.

All collected data were then securely transferred to an electronic database with regular quality assurance checks.

### Intervention compliance

Parent-reported compliance with spectacle wear was assessed through a structured telephone interview and a 24-hour diary. Parents were asked to estimate the total number of hours their child was awake and the total number of hours glasses were worn on a typical day. They reported a detailed time-log indicating the child’s activity (eg, asleep, feeding, awake) alongside whether glasses were on or off. Additional prompts explored typical routines for putting on the glasses and reasons for any periods of non-wear. Qualitative comments were recorded to capture contextual factors affecting compliance, such as the child removing glasses during feeding. See online supplemental appendix for the compliance interview form and example diary.

## Results

### Study population

**Figure 1:**
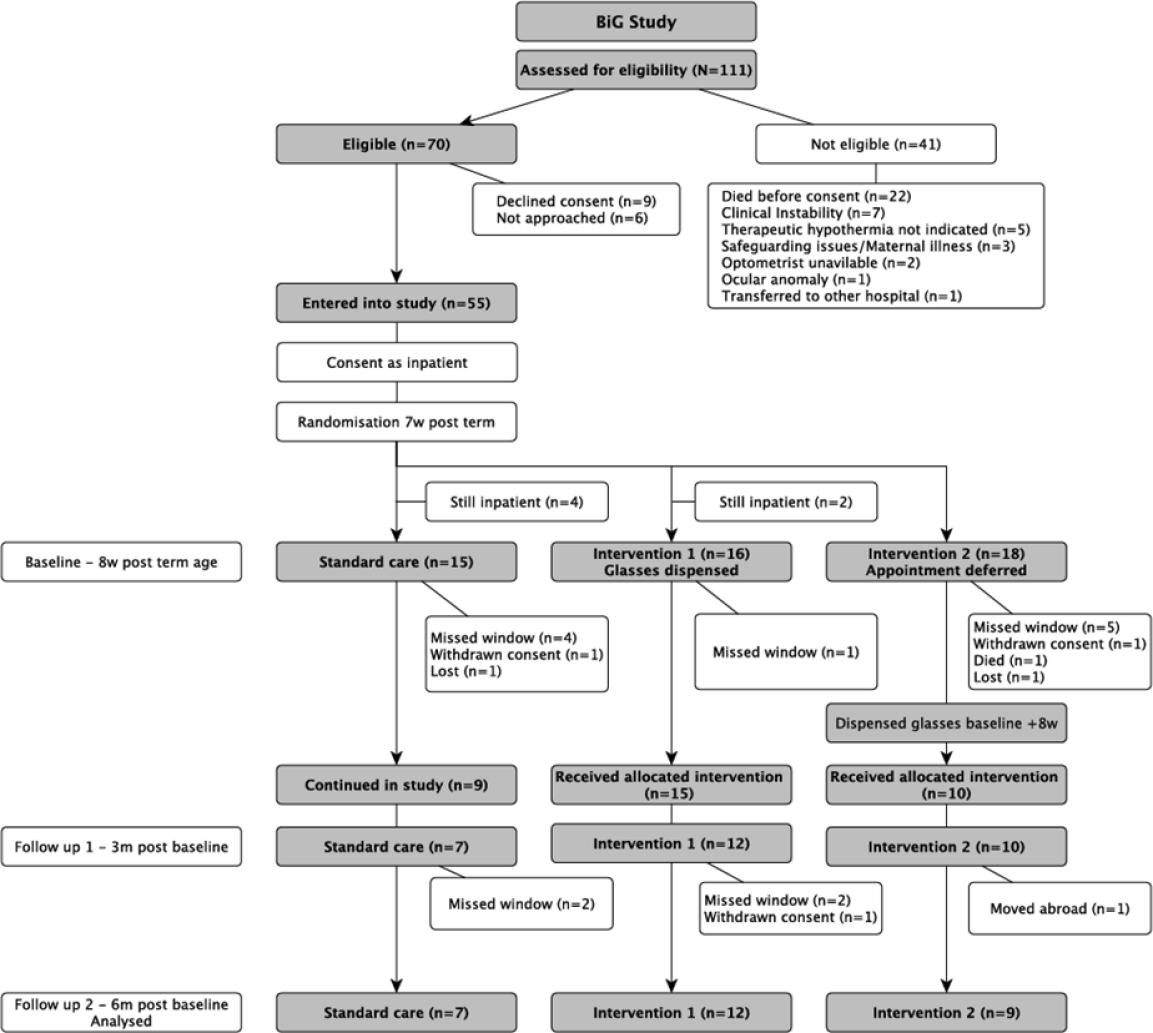
CONSORT diagram for BiG Feasibility Study. BIG, Babies in Glasses; CONSORT, Consolidated Standards of Reporting Trials.

Over the 12 months from 9 September 2021, 111 babies were born who met the study criteria, 102 babies at <29 weeks of gestation and 9 with HIE (figure 1). 41 were not included in the final study group because of infant death (n=22), clinical instability (n=7), not meeting criteria for therapeutic hypothermia (n=5) or other reasons. Of the 70 families eligible, 64 were approached. 55 consented to enter the trial (49 preterm; overall 79% of those eligible); families of 6 infants were not available for consent and there were 9 refusals.

19 were allocated to standard treatment and 18 to each of interventions 1 and 2, respectively, but 6 patients were not discharged home as anticipated, so they were excluded from the trial, and 15 others failed to attend the baseline assessment appointment. Overall, 34/55 (62%) were successfully enrolled: 9 continued with standard treatment, 15 were prescribed glasses at 8 weeks corrected age and 10 prescribed glasses at 16 weeks (table 1).

**Table 1:** Demographics, clinical outcomes and comorbidities by study arm

	Preterm group			HIE group		
	Standard care	Intervention 1	Intervention 2	Standard care	Intervention 1	Intervention 2
Male sex	11/17 (65%)	11/17 (65%)	7/15 (47%)	2/2 (100%)	0/1 (0%)	2/3 (67%)
Gestational age, mean (SD)	26 (2.0)	26 (1.9)	27 (1.6)	38.5 (0.7)	41 (-)	38.7 (2.1)
Birth weight (g), median (IQR)	795 (560–1170)	799 (575–1386)	890 (452–1290)	3520 (3290 –-)	3387 (---)	2852 (2800 –-)
Ethnicity						
White	3	5	5	1	0	1
Black	4	2	2	1	1	1
Asian	4	3	5	0	0	0
Mixed/other	6	7	3	0	0	1
Plurality						
Singleton	10	6	9	2	1	3
Twin	4	11	3	0	0	0
Triplet	3	0	3	0	0	0
Bronchopulmonary dysplasia*	8/17 (47%)	10/17 (59%)	5/15 (33%)			
Necrotising enterocolitis	3/17 (18%)	3/17 (18%)	1/15 (7%)			
Sepsis (n episodes)	3/17 (18%)	6/17 (35%)	4/15 (27%)			
Intraventricular haemorrhage	1/17 (6%)	2/17 (12%)	3/15 (20%)			
Periventricular leucomalacia	4/17 (24%)	8/17 (47%)	3/15 (20%)			
ROP†	1/17 (6%)	3/17 (18%)	0/15 (0%)			
Therapeutic hypothermia (days)				3.5	3	3 3
Seizures				0/2 (0%)	0/1 (0%)	1/3 (33%)
Abnormal MRI scan				2/2 (100%)	0/1 (0%)	2/3 (67%)

* Supplemental oxygen at 36 weeks postmenstrual age.

† ROP requiring treatment.

ROP, retinopathy of prematurity.

6-month postintervention outcomes were available for 28 infants (51% of those enrolled, but 82% of those who managed to attend baseline appointment), comprising 6, 11 and 6 preterm infants and 1, 1 and 3 HIE infants in groups A, B1 and B_2_, respectively. Eight dropouts after consent were for clinical reasons outside the study’s control (unplanned prolonged infant hospital stay or infant death), the remainder were missed appointments within the specified time window, withdrawal and in one case the family moved abroad and could not be followed up. Commencement of the study, recruitment and follow-up were compromised by restrictions imposed during the COVID-19 pandemic.

### Compliance with intervention

All children who entered the study were successfully dispensed glasses, except for one family who withdrew when allocation to glasses was revealed. Among the 28 infants and families that completed the trial, 57 (of 68, 84%) compliance telephone questionnaires were completed. Non-completion coincided mainly with maternity leave for the researcher, and 3 missed questionnaires were due to non-receipt of glasses (n=2) and parent unavailability (n=1).

The median daily wear times were similar in both intervention groups 1 and 2 (overall 2.0 hours/day; IQR 1–4 hours). Intervention 1 average group wear was 2 hours/day (IQR 1–4 hours) compared with intervention 2, where average group wear was 2 h/day (IQR 1.5–3 hours), indicating more consistent wear times across intervention 2.

### Clinical assessment

Improvements in mean LogMAR visual acuity from baseline to 6 months post-term age were standard care: 0.47 (95%CI 0.05 to 0.89); Intervention 1: corrected 0.66 (95% CI 0.34 to 0.98); Intervention 2: corrected 0.37 (95% CI 0.09 to 0.64) (table 2).

**Table 2:** Summary of uncorrected and corrected visual acuity, refractive error and accommodative lag (+)/lead (−) across study arms, comparing baseline to final visit measurements in the right eye

	Baseline			Visit 2 assessment (12-week follow-up)			Final assessment (24-week follow-up)		
	Standard care	Intervention 1	Intervention 2	Standard care	Intervention 1	Intervention 2	Standard care	Intervention 1	Intervention 2
Uncorrected logMAR (minimum angle of resolution) visual acuity Mean (SD)	1.48 (0.31) n=9	1.45 (0.33) n=14	1.09 (0.25) n=10	0.91 (0.15) n=7	1.08 (0.35) n=9	0.88 (0.15) n=10	0.96 (0.21) n=7	0.88 (0.45) n=10	0.76 (0.39) n=5
Corrected logMAR visual acuity Mean (SD)	–	–	–	–	0.89 (0.28) n=11	0.70 (0.17) n=7	–	0.79 (0.38) n=10	0.74 (0.34) n=8
Refractive error* (dioptres, D), Mean (SD)	+1.94 (2.07) n=9	+2.15 (1.43) n=10	+1.55 (1.29) n=10	+1.11 (1.44) n=7	+2.07 (1.25) n=7	+0.38 (3.14) n=10	+0.75 (1.21) n=7	+1.39 (1.61) n=7	−0.17 (3.60) n=9
Refractive error (D)† Mean (95% CI)	–	–	–	0.68 (−0.79 to 2.15) n=7	0.00 (−0.82 to 0.82) n=7	1.18 (−0.45 to 2.80) n=10	1.04 (−0.16 to 2.23) n=7	0.43 (−0.62 to 1.48) n=7	1.81 (−0.40 to 4.02) n=9
Uncorrected accommodative response (D) Mean (SD)	−0.25 (1.75) n=8	−0.42 (1.59) n=12	0.20 (1.51) n=10	0.04 (1.16) n=7	0.17 (1.30) n=12	1.00 (1.03) n=9	0.43 (0.59) n=7	−0.15 (0.98) n=10	0.03 (0.79) n=9

* Mean spherical equivalent right eye.

† Difference in means, baseline visit minus final visit, that is, myopic shift.

Among the 29 infants born <29 weeks of gestation, the equivalent figures were standard care: 0.35 (95% CI −0.02 to 0.72); Intervention 1: corrected 0.67 (95% CI 0.31 to 1.03); Intervention 2: corrected 0.37 (95% CI −0.03 to 0.72).

### Refractive error

Changes in refractive error demonstrated a normal pattern of emmetropisation, namely reduction in hyperopia observed from baseline to follow-up visits in all arms (table 2). There were no statistical or clinical differences observed between the three groups in terms of the amount of refractive change measured. One infant exhibited a shift from mildly myopic at baseline to approximately −8.50 DS at 12-week follow-up—prior to receipt or wear of corrective glasses. This infant was being monitored for active ROP.

### Functional vision

Infants completed the ADCDEFV at both baseline and the final assessment (table 3). Of interest was the improved rate of object permanence when fully covered in the intervention 1 group (56%) compared with Standard care (29%; OR 3.13 (95% CI 0.38 to 25.6), which was not as marked when glasses were prescribed at 16 weeks corrected age (44%). Fully covered object permanence refers to an infant’s ability to understand that an object continues to exist even when it is completely hidden from view.^23^

**Table 3:** Pass rates for ABCDEFV items assessed binocularly

	Baseline			Final assessment		
	Standard care	Intervention 1	Intervention 2	Standard care	Intervention 1	Intervention 2
	n=9	n=15	n=10	n=7 age 8 months*	n=11 age 8 months*	n=9 age 10 months*
Corneal reflections	3 (33%)	6 (40%)	8 (80%)	7 (100%)	8 (73%)	8 (89%)
Diffuse light reaction (RHS)	3 (38%)†	4 (29%)†	7 (78%)†	6 (100%)†	7 (88%)†	8 (100%)†
Lateral tracking (RHS)	6 (67%)	10 (71%)†	9 (90%)	7 (100%)	10 (100%)†	9 (100%)
Saccadic/smooth tracking (RHS)	6 (100%)†	10 (100%)†	9 (100%)†	7 (100%)	10 (100%)†	9 (100%)
Peripheral refixation (RHS)	5 (56%)	6 (43%)†	9 (90%)	7 (100%)	9 (90%)†	9 (100%)
Sustained visual attention (RHS)	1 (14%)†	2 (15%)†	3 (38%)†	5 (71%)	6 (67%)†	9 (100%)
Distance visual attention is broken	2 (29%)†	5 (39%)†	3 (38%)†	6 (86%)	7 (78%)†	9 (100%)
Pupillary response to light	8 (100%)†	12 (100%)†	10 (100%)†	7 (100%)	11 (100%)	9 (100%)
Object permanence (partially covered)	–	–	–	5 (71%)	8 (89%)†	7 (78%)
Object permanence (fully covered)	–	–	–	2 (29%)	5 (56%)†	4 (44%)
Batting and reaching	1 (11%)	0	4 (40%)	7 (100%)	10 (100%)†	9 (100%)
Follows fallen toy	–	–	1 (14%)†	6 (86%)	9 (90%)†	9 (100%)
Defensive blink	5 (56%)	9 (64%)†	10 (100%)	7 (100%)	10 (100%)†	9 (100%)
Convergence	8 (89%)	8 (53%)	10 (100%)	7 (100.0%)	10 (100%)†	9 (100%)

* Age corrected for preterm birth where appropriate (ie, post term age).

† Not all infants completed this task.

ABCDEFV, A Test Battery of Child Development for Examining Functional Vision; RHS, right hand side.

### Health economics

Provision of the intervention followed these steps: (1) babies measured by optometrist to fit glasses, prescription entered onto a form and sent to dispensing optician; (2) dispensing optician sends the order to the external laboratory; (3) lab makes glasses and sends glasses to dispensing optician, who then sends to the participant. In the case of this feasibility study, the optometrist acted as the dispensing optician due to staffing issues.

Costs included the price paid for the frames, lenses, postage and packaging for receiving the glasses from the lab, and staff time. Staff time has been costed here as band 7, according to the unit costs for hospital-based scientific and professional staff (PSSRU 2024, Table 11.1.2, £66 per working hour).^25^ It was considered by the optometrist that she spent around 10 min on measuring and completing the form, and around 10 min completing the order as acting dispensing optician, so a total of 20 min of staff time was included in the cost. Replacements were required for some babies due to outgrowing the frame and due to requiring prescription changes. There were no breakages during the study. Tables providing intervention cost information are in the online supplemental appendix.

Completion rates of the RUQ by parents were high. Details of use of services are given in the online supplemental appendix. Time off work by the parent completing the form was zero as they were on parental leave from work, and around a third of the respondents’ partners had taken time off work in the 3 months preceding baseline (with a mean (SD) of 21 (10.5) days off), around a quarter had taken time off between baseline and 3 months (mean (SD) of 26 (32) days off), and 7% had taken time off between 3 and 6 months (with a mean (SD) of 30 (0) days off). Smaller numbers of other family members also took time off. Full details are in the online supplemental appendix.

### Safety

No safety issues in terms of mechanical trauma were associated with the prescription and wearing of glasses in this study, confirmed by the Independent Data Monitoring Committee. A range of clinical adverse events was reported, mainly intercurrent illnesses, all unrelated to the intervention.

### Harms

No harms were identified throughout the study, and no instances of mechanical trauma or adverse effects related to the prescription of glasses were reported among participants.

## Discussion

We have demonstrated the feasibility of a study of early spectacle intervention in this vulnerable patient group. 55 of 70 (79%) eligible families consented to join the trial. Of these, 62% (n=34) were successfully enrolled on the study, dropouts being caused by intercurrent mortality after consent, prolonged inpatient stays and challenges for parents in attending extra outpatient appointments at the hospital—particularly those with multiple births. No safety issues were identified, and glasses were successfully dispensed after assessment for all infants in the intervention arms. Emmetropisation was, on average, 0.60D less over 6 months in the early glasses (compared with no glasses) group but, as for the possible benefit in acuity, which would more than compensate for this clinically, the CIs were wide. Also, this slightly higher hypermetropia in the treated group at 6 months might represent another benefit, as premature children (with or without ROP) are known to be at risk of myopia when older.^26^ Acuity assessment and functional assessment of vision revealed potential benefit for the Intervention 1 arm—those receiving glasses at 8 weeks post term demonstrated on average a greater improvement in visual acuity and were more likely to develop object permanence, an important developmental stage in vision and cognitive development; for instance, it might promote motor skills such as reaching.

This feasibility study was not powered to determine statistically significant benefit and the CIs for comparisons are thus wide. The parents were very keen to take part and found the wearing of glasses acceptable. Although there were no challenges with recruitment to the HIE stratum in this study, recent improvements in clinical protocols—and the associated reduction in the prevalence of HIE among term-born infants—may make recruitment to this group more difficult in future definitive studies compared with the preterm group. This, however, reflects positive progress in neonatal care and improved outcomes for affected infants. Furthermore, the benefit seemed marginally greater among the preterm versus the term HIE babies. Very preterm children have lifelong issues with vision and cognition and thus may benefit greatly from early normalisation of near vision. The present study suggests that this may be possible. The present study identifies the considerable challenge to parents of extra clinic visits and the wearing of glasses in small infants, and attention may be required to extend the windows during which visits can be made and/or introducing the option for home assessments. Combining research assessments with routine clinic visits is an option but one which was dispreferred by our PPI group who felt it would be too demanding for a young infant.

A potential criticism of the present study design is the administration of optical correction to infants who may not require it. The lack of standard assessments to determine CVI at young ages means that this is inevitable, although we consider that this is likely to be a minority of babies, and the benefits would outweigh the risks. Several studies identify significantly worse vision at later ages in both the clinical groups we enrolled compared with full term births without HIE. Hence, we carried out the feasibility study including those at highest risk. For example, nearly 2/3 of children may have poor visual acuity following HIE.^8 10 27^ Children born at very low gestational ages are at much higher risk of amblyopia, refractive error, strabismus and visuo-perceptual problems,^7 28 29^ which may be amenable to early correction with glasses.^15^ Furthermore, in our feasibility study, only two out of the seven babies in the untreated group had a measurable vision within the age-related norms at 8 months old.

## Conclusions

This is the first study to attempt to correct near vision in young infants. Early infancy is a critical period for visual development, during which typically developing infants already have limited ability to accommodate. While this coincides with a period of poor accommodation in typically developing infants, infants who have experienced a perinatal or neonatal brain injury are at even greater risk of impaired accommodation, making early intervention particularly important in this group.

The present study provides evidence that the prescription of near vision glasses to young infants at risk of perinatal brain insults is feasible and that outcomes may be enhanced compared with no glasses or when glasses are prescribed at 8 weeks post-term rather than at 16 weeks. Potential benefits in terms of visual acuity and cognitive development have been demonstrated. All vision assessments conducted in the feasibility study will be included in the final RCT, encompassing visual acuity measures and the full array of ABCDEFV developmental assessments, along with a shortened version of the RUQ, as some items were not used by any participants.

In this feasibility study, we included both infants born very preterm and those who developed HIE after birth at full term. Preterm babies comprised the large majority of study recruits (29/35) and are much more common in the neonatal population, which has also been the experience of other recent trials (eg, the DOLFIN Trial NIHR130925).^30^ The preterm group also showed greater potential benefit from glasses in subgroup analysis, with intervention 1 showing average acuity gains of nearly three lines more on the acuity chart than those measured in the standard care group. Studying very preterm populations has the advantage of a more homogeneous population, with established follow-up protocols (NICE Guideline NG72 2017).^31^ Hence, we plan a definitive RCT focusing solely on infants born at <30 weeks gestation and offering glasses at 8 weeks.

## Supplementary material

10.1136/bmjopen-2025-107894 online supplemental file 1

## Data Availability

Data are available on reasonable request.
