# Supplementary material for: Can provision of near vision glasses as an early intervention improve visual outcomes in infants at risk of perinatal brain insult? The Babies in Glasses (BiG) randomised feasibility trial
Source: BMJ Open. 2026 Feb 16;16(2):e107894. doi: 10.1136/bmjopen-2025-107894 (PMC12911751; doi:10.1136/bmjopen-2025-107894)
Supplement: online supplemental file 1 [file bmjopen-16-2-s001.docx]

**Appendix**

**Health Economics**

**Appendix A. Health care resources reported by study participants for the 3 months preceding baseline, by arm.**

|  | **Arm A** | **Arm B1** | **Arm B2** | **Total** |
| --- | --- | --- | --- | --- |
| **Numbers of visits or attendances reported** | 9 | 13 | 10 | 32 |
| **Hospital admissions and visits to A&E** |  |  |  |  |
| **Was child sent home with oxygen?** |  |  |  |  |
| Yes, n (%) | 0 (0%) | 4 (31%) | 1 (10%) | 5 (16%) |
| No, n (%) | 9 (100%) | 9 (69%) | 9 (90%) | 27 (84%) |
| Missing, n (%) | 0 (0%) | 0 (0%) | 0 (0%) | 0 (0%) |
| If yes, mean (SD) days from discharge to last receipt |  | 6.7 (3.1) | 0 (0) | 5 (4.2) |
| If yes, still taking supplemental oxygen? |  |  |  |  |
| Yes, n (%) |  | 3 (75%) | 1 (100%) | 4 (80%) |
| No, n (%) |  | 0 (0%) | 0 (0%) | 0 (0%) |
| Missing, n (%) |  | 1 (25%) | 0 (0%) | 1 (20%) |
| **Has child been to A&E in last 3 months?** |  |  |  |  |
| Yes, n (%); mean (SD) visits per patient | 1 (11%); 1 (0) | 2 (15%); 2 (1.4) | 5 (50%); 1.2 (0.4) | 8 (25%); 1.4 (0.7) |
| No, n (%) | 8 (89%) | 10 (77%) | 5 (50%) | 23 (72%) |
| Missing, n (%) | 0 (0%) | 1 (8%) | 0 (0%) | 1 (3%) |
| If yes, mean (SD) nights per visit | 0 (0) | 0.5 (0.7) | 0.2 (0.4) | 0.3 (0.5) |
| **Has child been admitted to hospital in last 3 months?** |  |  |  |  |
| Yes, n (%); mean (SD) visits per patient | 1 (11%); 1 (0) | 0 (0%); 0 (0) | 0 (0%); 0 (0) | 1 (3%); 1 (0) |
| **Has child attended hospital as day patient for procedure or test in last 3 months?** |  |  |  |  |
| Yes, n (%); mean (SD) visits per patient | 0 (0%); 0 (0) | 2 (15%); 1 (0) | 1 (10%); 1 (0) | 3 (9%); 1 (0) |
| **Has child had outpatient appointment in last 3 months?** |  |  |  |  |
| Yes, n (%) | 7 (78%) | 9 (69%) | 10 (100%) | 26 (81%) |
| Neurodevelopmental Assessments |  |  |  |  |
| Yes, n (%); mean (SD) visits per patient | 1 (11%); 1 (0) | 2 (15%); 1 (0) | 10 (100%); 1.3 (0.5) | 13 (41%); 1.2 (0.4) |
| Speech and language therapist |  |  |  |  |
| Yes, n (%); mean (SD) visits per patient | 1 (11%); 3 (0) | 3 (23%); 1.7 (1.2) | 3 (30%); 1.3 (0.6) | 7 (22%); 1.7 (1) |
| Physiotherapist |  |  |  |  |
| Yes, n (%); mean (SD) visits per patient | 0 (0%); 0 (0) | 1 (8%); 1 (0) | 4 (40%); 2.3 (0.5) | 5 (16%); 2 (0.7) |
| Occupational therapist |  |  |  |  |
| Yes, n (%); mean (SD) visits per patient | 0 (0%); 0 (0) | 3 (23%); 1.3 (0.6) | 0 (0%); 0 (0) | 3 (9%); 1.3 (0.6) |
| Dietician/feeding clinic |  |  |  |  |
| Yes, n (%); mean (SD) visits per patient | 3 (33%); 1.7 (1.2) | 2 (15%); 2.5 (0.7) | 5 (50%); 1.4 (0.9) | 10 (31%); 1.7 (0.9) |
| Audiologist |  |  |  |  |
| Yes, n (%); mean (SD) visits per patient | 1 (11%); 1 (0) | 2 (15%); 1.5 (0.7) | 1 (10%); 1 (0) | 4 (13%); 1.3 (0.5) |
| Respiratory specialist |  |  |  |  |
| Yes, n (%); mean (SD) visits per patient | 0 (0%); 0 (0) | 0 (0%); 0 (0) | 1 (10%); 1 (0) | 1 (3%); 1 (0) |
| Ophthalmology |  |  |  |  |
| Yes, n (%); mean (SD) visits per patient | 4 (44%); 2.5 (1.7) | 5 (38%); 2 (1.4) | 3 (30%); 8 (10.4) | 12 (38%); 3.7 (5.3) |
| Other |  |  |  |  |
| Yes, n (%); mean (SD) visits per patient | 0 (0%); 0 (0) | 0 (0%); 0 (0) | 2 (20%); 1 (0) | 2 (6%); 1 (0) |
| Other, Cardiology |  |  |  |  |
| Yes, n (%); mean (SD) visits per patient | 0 (0%); 0 (0) | 0 (0%); 0 (0) | 1 (10%); 1 (0) | 1 (3%); 1 (0) |
| Other, Haematology |  |  |  |  |
| Yes, n (%); mean (SD) visits per patient | 0 (0%); 0 (0) | 0 (0%); 0 (0) | 1 (10%); 6 (0) | 1 (3%); 6 (0) |
| Other, Paediatrician/Neonatal |  |  |  |  |
| Yes, n (%); mean (SD) visits per patient | 0 (0%); 0 (0) | 0 (0%); 0 (0) | 0 (0%); 0 (0) | 0 (0%); 0 (0) |
| **Has child used community services in last 3 months?** |  |  |  |  |
| Yes, n (%) | 9 (100%) | 12 (92%) | 10 (100%) | 31 (97%) |
| GP - Yes, n (%) | 8 (89%) | 10 (77%) | 8 (80%) | 26 (81%) |
| GP, mean (SD) clinic visits | 1.4 (0.7) | 1.3 (0.5) | 1.4 (1.2) | 1.3 (0.8) |
| Practice nurse - Yes, n (%) | 4 (44%) | 4 (31%) | 5 (50%) | 13 (41%) |
| Practice nurse, mean (SD) clinic visits | 1.5 (1.3) | 1.5 (1.3) | 7.8 (13.6) | 3.9 (8.5) |
| Practice nurse, mean (SD) home visits | 0.5 (0.6) | 1.5 (3) | 0 (0) | 0.6 (1.7) |
| Practice nurse, mean (SD) phone/video contacts | 0 (0) | 0 (0) | 0 (0) | 0 (0) |
| Health visitor - Yes, n (%) | 6 (67%) | 8 (62%) | 8 (80%) | 22 (69%) |
| Health visitor, mean (SD) clinic visits | 0.3 (0.8) | 0.3 (0.5) | 0.6 (1.1) | 0.4 (0.8) |
| Health visitor, mean (SD) home visits | 1.2 (1.2) | 1.4 (1.2) | 2.3 (1.2) | 1.6 (1.2) |
| Health visitor, mean (SD) phone/video contacts | 0.2 (0.4) | 0 (0) | 0 (0) | 0 (0.2) |
| Comm Paediatrician - Yes, n (%) | 0 (0%) | 0 (0%) | 1 (10%) | 1 (3%) |
| Comm Paediatrician, mean (SD) clinic visits | 0 (0) | 0 (0) | 1 (0) | 1 (0) |
| Comm Paediatrician, mean (SD) home visits | 0 (0) | 0 (0) | 0 (0) | 0 (0) |
| Comm Paediatrician, mean (SD) phone/video contacts | 0 (0) | 0 (0) | 0 (0) | 0 (0) |
| Social worker - Yes, n (%) | 0 (0%) | 0 (0%) | 1 (10%) | 1 (3%) |
| Social worker, mean (SD) clinic visits | 0 (0) | 0 (0) | 0 (0) | 0 (0) |
| Social worker, mean (SD) home visits | 0 (0) | 0 (0) | 0 (0) | 0 (0) |
| Social worker, mean (SD) phone/video contacts | 0 (0) | 0 (0) | 1 (0) | 1 (0) |
| OT - Yes, n (%) | 0 (0%) | 1 (8%) | 0 (0%) | 1 (3%) |
| OT, mean (SD) clinic visits | 0 (0) | 1 (0) | 0 (0) | 1 (0) |
| OT, mean (SD) home visits | 0 (0) | 0 (0) | 0 (0) | 0 (0) |
| OT, mean (SD) phone/video contacts | 0 (0) | 0 (0) | 0 (0) | 0 (0) |
| SALT - Yes, n (%) | 1 (11%) | 0 (0%) | 0 (0%) | 1 (3%) |
| SALT, mean (SD) clinic visits | 1 (0) | 0 (0) | 0 (0) | 1 (0) |
| SALT, mean (SD) home visits | 0 (0) | 0 (0) | 0 (0) | 0 (0) |
| SALT, mean (SD) phone/video contacts | 0 (0) | 0 (0) | 0 (0) | 0 (0) |
| Physio - Yes, n (%) | 0 (0%) | 2 (15%) | 0 (0%) | 2 (6%) |
| Physio, mean (SD) clinic visits | 0 (0) | 2.5 (0.7) | 0 (0) | 2.5 (0.7) |
| Physio, mean (SD) home visits | 0 (0) | 0 (0) | 0 (0) | 0 (0) |
| Physio, mean (SD) phone/video contacts | 0 (0) | 0 (0) | 0 (0) | 0 (0) |
| Dietician/feeding - Yes, n (%) | 0 (0%) | 2 (15%) | 0 (0%) | 2 (6%) |
| Dietician/feeding, mean (SD) clinic visits | 0 (0) | 2.5 (2.1) | 0 (0) | 2.5 (2.1) |
| Dietician/feeding, mean (SD) home visits | 0 (0) | 0 (0) | 0 (0) | 0 (0) |
| Dietician/feeding, mean (SD) phone/video contacts | 0 (0) | 0 (0) | 0 (0) | 0 (0) |
| Osteopath - Yes, n (%) | 0 (0%) | 0 (0%) | 1 (10%) | 1 (3%) |
| Osteopath, mean (SD) clinic visits | 0 (0) | 0 (0) | 4 (0) | 4 (0) |
| Osteopath, mean (SD) home visits | 0 (0) | 0 (0) | 0 (0) | 0 (0) |
| Osteopath, mean (SD) phone/video contacts | 0 (0) | 0 (0) | 50 (0) | 50 (0) |
| Other - Yes, n (%) | 0 (0%) | 1 (8%) | 0 (0%) | 1 (3%) |
| Other, mean (SD) times seen | 0 (0) | 1 (0) | 0 (0) | 1 (0) |
| Other, n (%) paid for | 0 (0%) | 1 (8%) | 0 (0%) | 1 (3%) |
| Other, mean (SD) price per visit | 0 (0) | 0 (0) | 0 (0) | 0 (0) |
| **Has child taken medications in last 3 months?** |  |  |  |  |
| Yes, n (%) | 4 (44%) | 12 (92%) | 7 (70%) | 23 (72%) |

**Appendix B. Health care resources reported by study participants for the 3-month period between baseline and 3m visits, by arm.**

|  | **Arm A** | **Arm B1** | **Arm B2** | **Total** |
| --- | --- | --- | --- | --- |
| **Numbers of visits or attendances reported** | 7 | 12 | 10 | 29 |
| **Hospital admissions and visits to A&E** |  |  |  |  |
| **Was child sent home with oxygen?** |  |  |  |  |
| Yes, n (%) | 0 (0%) | 3 (25%) | 1 (10%) | 4 (14%) |
| No, n (%) | 7 (100%) | 8 (67%) | 9 (90%) | 24 (83%) |
| Missing, n (%) | 0 (0%) | 1 (8%) | 0 (0%) | 1 (3%) |
| If yes, mean (SD) days from discharge to last receipt |  | 14.7 (13.6) | 59 (0) | 25.8 (24.8) |
| If yes, still taking supplemental oxygen? |  |  |  |  |
| Yes, n (%) |  | 2 (66.67%) | 0 (0%) | 2 (50%) |
| No, n (%) |  | 1 (33.33%) | 1 (100%) | 2 (50%) |
| Missing, n (%) |  | 0 (0%) | 0 (0%) | 0 (0%) |
| **Has child been to A&E in last 3 months?** |  |  |  |  |
| Yes, n (%); mean (SD) visits per patient | 2 (29%); 1 (0) | 3 (25%); 2.3 (1.2) | 4 (40%); 1 (0) | 9 (31%); 1.4 (0.9) |
| If yes, mean (SD) nights per visit | 0 (0) | 0 (0) | 0.3 (0.5) | 0.1 (0.3) |
| **Has child been admitted to hospital in last 3 months?** |  |  |  |  |
| Yes, n (%); mean (SD) visits per patient | 1 (14%); 1 (0) | 1 (8%); 1 (0) | 0 (0%); 0 (0) | 2 (7%); 1 (0) |
| If yes, mean (SD) nights per visit | 1 (0) | 7 (0) |  | 4 (4.2) |
| **Has child attended hospital as day patient for procedure or test in last 3 months?** |  |  |  |  |
| Yes, n (%); mean (SD) visits per patient | 0 (0%); 0 (0) | 0 (0%); 0 (0) | 0 (0%); 0 (0) | 0 (0%); 0 (0) |
| **Has child had outpatient appointment in last 3 months?** |  |  |  |  |
| Yes, n (%) | 5 (71%) | 9 (75%) | 10 (100%) | 24 (83%) |
| Neurodevelopmental Assessments |  |  |  |  |
| Yes, n (%); mean (SD) visits per patient | 4 (57%); 1.3 (0.5) | 8 (67%); 1 (0) | 8 (80%); 1 (0) | 20 (69%); 1.1 (0.2) |
| Speech and language therapist |  |  |  |  |
| Yes, n (%); mean (SD) visits per patient | 1 (14%); 1 (0) | 1 (8%); 1 (0) | 5 (50%); 1.2 (0.4) | 7 (24%); 1.1 (0.4) |
| Physiotherapist |  |  |  |  |
| Yes, n (%); mean (SD) visits per patient | 0 (0%); 0 (0) | 0 (0%); 0 (0) | 2 (20%); 4.5 (2.1) | 2 (7%); 4.5 (2.1) |
| Occupational therapist |  |  |  |  |
| Yes, n (%); mean (SD) visits per patient | 0 (0%); 0 (0) | 1 (8%); 5 (0) | 0 (0%); 0 (0) | 1 (3%); 5 (0) |
| Dietician/feeding clinic |  |  |  |  |
| Yes, n (%); mean (SD) visits per patient | 2 (29%); 1 (0) | 3 (25%); 1 (0) | 5 (50%); 1.2 (0.4) | 10 (34%); 1.1 (0.3) |
| Audiologist |  |  |  |  |
| Yes, n (%); mean (SD) visits per patient | 0 (0%); 0 (0) | 1 (8%); 1 (0) | 0 (0%); 0 (0) | 1 (3%); 1 (0) |
| Respiratory specialist |  |  |  |  |
| Yes, n (%); mean (SD) visits per patient | 0 (0%); 0 (0) | 0 (0%); 0 (0) | 0 (0%); 0 (0) | 0 (0%); 0 (0) |
| Ophthalmology |  |  |  |  |
| Yes, n (%); mean (SD) visits per patient | 1 (14%); 1 (0) | 3 (25%); 3 (2.6) | 3 (30%); 2 (1) | 7 (24%); 2.3 (1.8) |
| Other |  |  |  |  |
| Yes, n (%); mean (SD) visits per patient | 0 (0%); 0 (0) | 1 (8%); 0 (0) | 2 (20%); 1 (0) | 3 (10%); 1 (0) |
| **Has child used community services in last 3 months?** |  |  |  |  |
| Yes, n (%) | 6 (86%) | 9 (75%) | 5 (50%) | 20 (69%) |
| GP - Yes, n (%) | 3 (43%) | 4 (33%) | 5 (50%) | 12 (41%) |
| GP, mean (SD) clinic visits | 1.7 (0.6) | 1.8 (0.5) | 1.4 (0.5) | 1.6 (0.5) |
| Practice nurse - Yes, n (%) | 2 (29%) | 2 (17%) | 1 (10%) | 5 (17%) |
| Practice nurse, mean (SD) clinic visits | 2 (1.4) | 1 (1.4) | 0 (0) | 1.2 (1.3) |
| Practice nurse, mean (SD) home visits | 0 (0) | 0.5 (0.7) | 4 (0) | 1 (1.7) |
| Practice nurse, mean (SD) phone/video contacts | 0 (0) | 0 (0) | 0 (0) | 0 (0) |
| Health visitor - Yes, n (%) | 3 (43%) | 6 (50%) | 1 (10%) | 10 (34%) |
| Health visitor, mean (SD) clinic visits | 2 (3.5) | 0.3 (0.8) | 0 (0) | 0.8 (1.9) |
| Health visitor, mean (SD) home visits | 1 (1.7) | 1.7 (1.4) | 1 (0) | 1.4 (1.3) |
| Health visitor, mean (SD) phone/video contacts | 0.3 (0.6) | 0 (0) | 0 (0) | 0.1 (0.3) |
| Comm Paediatrician - Yes, n (%) | 0 (0%) | 2 (17%) | 0 (0%) | 2 (7%) |
| Comm Paediatrician, mean (SD) clinic visits | 0 (0) | 0.5 (0.7) | 0 (0) | 0.5 (0.7) |
| Comm Paediatrician, mean (SD) home visits | 0 (0) | 1.5 (2.1) | 0 (0) | 1.5 (2.1) |
| Comm Paediatrician, mean (SD) phone/video contacts | 0 (0) | 0 (0) | 0 (0) | 0 (0) |
| Social worker - Yes, n (%) | 0 (0%) | 0 (0%) | 0 (0%) | 0 (0%) |
| Social worker, mean (SD) clinic visits | 0 (0) | 0 (0) | 0 (0) | 0 (0) |
| Social worker, mean (SD) home visits | 0 (0) | 0 (0) | 0 (0) | 0 (0) |
| Social worker, mean (SD) phone/video contacts | 0 (0) | 0 (0) | 0 (0) | 0 (0) |
| OT - Yes, n (%) | 0 (0%) | 1 (8%) | 0 (0%) | 1 (3%) |
| OT, mean (SD) clinic visits | 0 (0) | 2 (0) | 0 (0) | 2 (0) |
| OT, mean (SD) home visits | 0 (0) | 0 (0) | 0 (0) | 0 (0) |
| OT, mean (SD) phone/video contacts | 0 (0) | 0 (0) | 0 (0) | 0 (0) |
| SALT - Yes, n (%) | 1 (14%) | 1 (8%) | 1 (10%) | 3 (10%) |
| SALT, mean (SD) clinic visits | 3 (0) | 1 (0) | 3 (0) | 2.3 (1.2) |
| SALT, mean (SD) home visits | 0 (0) | 0 (0) | 0 (0) | 0 (0) |
| SALT, mean (SD) phone/video contacts | 0 (0) | 0 (0) | 0 (0) | 0 (0) |
| Physio - Yes, n (%) | 1 (14%) | 1 (8%) | 1 (10%) | 3 (10%) |
| Physio, mean (SD) clinic visits | 3 (0) | 12 (0) | 3 (0) | 6 (5.2) |
| Physio, mean (SD) home visits | 0 (0) | 0 (0) | 0 (0) | 0 (0) |
| Physio, mean (SD) phone/video contacts | 0 (0) | 0 (0) | 0 (0) | 0 (0) |
| Dietician/feeding - Yes, n (%) | 1 (14%) | 0 (0%) | 1 (10%) | 2 (7%) |
| Dietician/feeding, mean (SD) clinic visits | 3 (0) | 0 (0) | 1 (0) | 2 (1.4) |
| Dietician/feeding, mean (SD) home visits | 0 (0) | 0 (0) | 0 (0) | 0 (0) |
| Dietician/feeding, mean (SD) phone/video contacts | 0 (0) | 0 (0) | 0 (0) | 0 (0) |
| Osteopath - Yes, n (%) | 0 (0%) | 0 (0%) | 0 (0%) | 0 (0%) |
| Osteopath, mean (SD) clinic visits | 0 (0) | 0 (0) | 0 (0) | 0 (0) |
| Osteopath, mean (SD) home visits | 0 (0) | 0 (0) | 0 (0) | 0 (0) |
| Osteopath, mean (SD) phone/video contacts | 0 (0) | 0 (0) | 0 (0) | 0 (0) |
| Other - Yes, n (%) | 0 (0%) | 1 (8%) | 0 (0%) | 1 (3%) |
| Other, mean (SD) times seen | 0 (0) | 2 (0) | 0 (0) | 2 (0) |
| Other, n (%) paid for | 0 (0%) | 1 (8%) | 0 (0%) | 1 (3%) |
| Other, mean (SD) price per visit | 0 (0) | 0 (0) | 0 (0) | 0 (0) |
| **Has child taken medications in last 3 months?** |  |  |  |  |
| Yes, n (%) | 2 (29%) | 9 (75%) | 5 (50%) | 16 (55%) |

**Appendix C. Health care resources reported by study participants for the 3-month period between 3m and 6m visits, by arm.**

|  | **Arm A** | **Arm B1** | **Arm B2** | **Total** |
| --- | --- | --- | --- | --- |
| **Numbers of visits or attendances reported** | 7 | 1 | 9 | 27 |
| **Hospital admissions and visits to A&E** |  |  |  |  |
| **Was child sent home with oxygen?** |  |  |  |  |
| Yes, n (%) | 0 (0%) | 2 (18%) | 1 (11%) | 3 (11%) |
| No, n (%) | 7 (100%) | 8 (73%) | 8 (89%) | 23 (82%) |
| Missing, n (%) | 0 (0%) | 1 (9%) | 0 (0%) | 2 (7%) |
| If yes, mean (SD) days from discharge to last receipt |  | 102.5 (7.8) | 142 (0) | 115.7 (23.5) |
| If yes, still taking supplemental oxygen? |  |  |  |  |
| Yes, n (%) |  | 0 (0%) | 0 (0%) | 0 (0%) |
| No, n (%) |  | 2 (100%) | 1 (100%) | 3 (100%) |
| Missing, n (%) |  | 0 (0%) | 0 (0%) | 0 (0%) |
| **Has child been to A&E in last 3 months?** |  |  |  |  |
| Yes, n (%); mean (SD) visits per patient | 3 (43%); 1 (0) | 1 (9%); 1 (0) | 0 (0%); 0 (0) | 4 (14%); 1 (0) |
| No, n (%) | 4 (57%) | 9 (82%) | 9 (100%) | 22 (79%) |
| Missing, n (%) | 0 (0%) | 1 (9%) | 0 (0%) | 2 (7%) |
| If yes, mean (SD) nights per visit | 0.3 (0.6) | 0 (0) |  | 0.3 (0.5) |
| **Has child been admitted to hospital in last 3 months?** |  |  |  |  |
| Yes, n (%); mean (SD) visits per patient | 1 (14%); 1 (0) | 0 (0%); 0 (0) | 0 (0%); 0 (0) | 1 (4%); 1 (0) |
| If yes, mean (SD) nights per visit | 1 (0) |  |  | 1 (0) |
| **Has child attended hospital as day patient for procedure or test in last 3 months?** |  |  |  |  |
| Yes, n (%); mean (SD) visits per patient | 0 (0%); 0 (0) | 0 (0%); 0 (0) | 1 (11%); 1 (0) | 2 (7%); 1 (0) |
| **Has child had outpatient appointment in last 3 months?** |  |  |  |  |
| Yes, n (%) | 6 (86%) | 10 (91%) | 7 (78%) | 23 (82%) |
| Neurodevelopmental Assessments |  |  |  |  |
| Yes, n (%); mean (SD) visits per patient | 6 (86%); 1 (0) | 10 (91%); 1 (0) | 5 (56%); 1 (0) | 21 (75%); 1 (0) |
| Speech and language therapist |  |  |  |  |
| Yes, n (%); mean (SD) visits per patient | 1 (14%); 1 (0) | 3 (27%); 1 (0) | 0 (0%); 0 (0) | 4 (14%); 1 (0) |
| Physiotherapist |  |  |  |  |
| Yes, n (%); mean (SD) visits per patient | 0 (0%); 0 (0) | 0 (0%); 0 (0) | 1 (11%); 1 (0) | 1 (4%); 1 (0) |
| Occupational therapist |  |  |  |  |
| Yes, n (%); mean (SD) visits per patient | 0 (0%); 0 (0) | 0 (0%); 0 (0) | 0 (0%); 0 (0) | 0 (0%); 0 (0) |
| Dietician/feeding clinic |  |  |  |  |
| Yes, n (%); mean (SD) visits per patient | 1 (14%); 1 (0) | 4 (365); 1.3 (0.5) | 4 (44%); 1.3 (0.5) | 9 (32%); 1.2 (0.4) |
| Audiologist |  |  |  |  |
| Yes, n (%); mean (SD) visits per patient | 0 (0%); 0 (0) | 0 (0%); 0 (0) | 0 (0%); 0 (0) | 0 (0%); 0 (0) |
| Respiratory specialist |  |  |  |  |
| Yes, n (%); mean (SD) visits per patient | 0 (0%); 0 (0) | 0 (0%); 0 (0) | 0 (0%); 0 (0) | 0 (0%); 0 (0) |
| Ophthalmology |  |  |  |  |
| Yes, n (%); mean (SD) visits per patient | 1 (14%); 1 (0) | 0 (0%); 0 (0) | 1 (11%); 1 (0) | 2 (7%); 1 (0) |
| Other |  |  |  |  |
| Yes, n (%); mean (SD) visits per patient | 0 (0%); 0 (0) | 0 (0%); 0 (0) | 1 (11%); 0 (0) | 1 (4%); 0 (0) |
| **Has child used community services in last 3 months?** |  |  |  |  |
| Yes, n (%) | 3 (43%) | 6 (50%) | 5 (56%) | 14 (50%) |
| GP - Yes, n (%) | 2 (29%) | 3 (25%) | 1 (11%) | 6 (21%) |
| GP, mean (SD) clinic visits | 1 (0) | 1 (0) | 1 (0) | 1 (0) |
| GP, mean (SD) home visits | 0 (0) | 0 (0) | 0 (0) | 0 (0) |
| GP, mean (SD) phone/video contacts | 0 (0) | 0 (0) | 0 (0) | 0 (0) |
| Practice nurse - Yes, n (%) | 1 (14%) | 0 (0%) | 2 (22%) | 3 (11%) |
| Practice nurse, mean (SD) clinic visits | 1 (0) | 0 (0) | 1 (0) | 1 (0) |
| Practice nurse, mean (SD) home visits | 0 (0) | 0 (0) | 0 (0) | 0 (0) |
| Practice nurse, mean (SD) phone/video contacts | 0 (0) | 0 (0) | 0 (0) | 0 (0) |
| Health visitor - Yes, n (%) | 1 (14%) | 3 (27%) | 1 (11%) | 5 (18%) |
| Health visitor, mean (SD) clinic visits | 4 (0) | 0 (0) | 1 (0) | 1 (1.7) |
| Health visitor, mean (SD) home visits | 0 (0) | 1 (0) | 0 (0) | 0.6 (0.5) |
| Health visitor, mean (SD) phone/video contacts | 0 (0) | 0 (0) | 0 (0) | 0 (0) |
| Comm Paediatrician - Yes, n (%) | 0 (0%) | 0 (0%) | 0 (0%) | 0 (0%) |
| Comm Paediatrician, mean (SD) clinic visits | 0 (0) | 0 (0) | 0 (0) | 0 (0) |
| Comm Paediatrician, mean (SD) home visits | 0 (0) | 0 (0) | 0 (0) | 0 (0) |
| Comm Paediatrician, mean (SD) phone/video contacts | 0 (0) | 0 (0) | 0 (0) | 0 (0) |
| Social worker - Yes, n (%) | 0 (0%) | 0 (0%) | 0 (0%) | 0 (0%) |
| Social worker, mean (SD) clinic visits | 0 (0) | 0 (0) | 0 (0) | 0 (0) |
| Social worker, mean (SD) home visits | 0 (0) | 0 (0) | 0 (0) | 0 (0) |
| Social worker, mean (SD) phone/video contacts | 0 (0) | 0 (0) | 0 (0) | 0 (0) |
| OT - Yes, n (%) | 0 (0%) | 2 (18%) | 1 (11%) | 3 (11%) |
| OT, mean (SD) clinic visits | 0 (0) | 2 (1.4) | 1 (0) | 1.7 (1.2) |
| OT, mean (SD) home visits | 0 (0) | 0 (0) | 0 (0) | 0 (0) |
| OT, mean (SD) phone/video contacts | 0 (0) | 0 (0) | 0 (0) | 0 (0) |
| SALT - Yes, n (%) | 0 (0%) | 1 (9%) | 1 (11%) | 2 (7%) |
| SALT, mean (SD) clinic visits | 0 (0) | 1 (0) | 1 (0) | 1 (0) |
| SALT, mean (SD) home visits | 0 (0) | 0 (0) | 0 (0) | 0 (0) |
| SALT, mean (SD) phone/video contacts | 0 (0) | 0 (0) | 0 (0) | 0 (0) |
| Physio - Yes, n (%) | 1 (14%) | 1 (9%) | 2 (22%) | 4 (14%) |
| Physio, mean (SD) clinic visits | 3 (0) | 12 (0) | 1.5 (0.7) | 4.5 (5.1) |
| Physio, mean (SD) home visits | 0 (0) | 0 (0) | 0 (0) | 0 (0) |
| Physio, mean (SD) phone/video contacts | 0 (0) | 0 (0) | 0 (0) | 0 (0) |
| Dietician/feeding - Yes, n (%) | 0 (0%) | 0 (0%) | 0 (0%) | 0 (0%) |
| Dietician/feeding, mean (SD) clinic visits | 0 (0) | 0 (0) | 0 (0) | 0 (0) |
| Dietician/feeding, mean (SD) home visits | 0 (0) | 0 (0) | 0 (0) | 0 (0) |
| Dietician/feeding, mean (SD) phone/video contacts | 0 (0) | 0 (0) | 0 (0) | 0 (0) |
| Osteopath - Yes, n (%) | 0 (0%) | 0 (0%) | 0 (0%) | 0 (0%) |
| Osteopath, mean (SD) clinic visits | 0 (0) | 0 (0) | 0 (0) | 0 (0) |
| Osteopath, mean (SD) home visits | 0 (0) | 0 (0) | 0 (0) | 0 (0) |
| Osteopath, mean (SD) phone/video contacts | 0 (0) | 0 (0) | 0 (0) | 0 (0) |
| Other - Yes, n (%) | 0 (0%) | 2 (18%) | 1 (11%) | 3 (11%) |
| **Has child taken medications in last 3 months?** |  |  |  |  |
| Yes, n (%) | 2 (29%) | 8 (73%) | 5 (56%) | 15 (54%) |

**Appendix D. Time take off work by parent accompanying child, partner, and other family members: in the 3-month periods preceding baseline, 3-month timepoint and 6-month timepoint, by arm and overall.**

| **BASELINE**  **Have parents/family taken time off work in last 3 months due to child's health?** | **Arm A** | **Arm B1** | **Arm B2** | **Total** |
| --- | --- | --- | --- | --- |
| Have you taken time off? |  |  |  |  |
| Yes, n (%); mean (SD) no. days | 0 (0%); 0 (0) | 0 (0%); 0 (0) | 0 (0%); 0 (0) | 0 (0%); 0 (0) |
| No, n (%) | 9 (100%) | 12 (92%) | 10 (100%) | 31 (97%) |
| Missing, n (%) | 0 (0%) | 1 (8%) | 0 (0%) | 1 (3%) |
|  |  |  |  |  |
| Has your partner taken time off? |  |  |  |  |
| Yes, n (%); mean (SD) no. days | 2 (22%); 22 (11.3) | 6 (46%); 40.2 (39.4) | 3 (30%); 10.3 (6.4) | 11 (34%); 28.7 (31.4) |
| No, n (%) | 6 (67%) | 6 (46%) | 7 (70%) | 19 (59%) |
| Missing, n (%) | 0 (0%) | 1 (8%) | 0 (0%) | 1 (3%) |
|  |  |  |  |  |
| Have other family members taken time off? |  |  |  |  |
| Yes, n (%); mean (SD) no. days | 1 (11%); 30 (0) | 2 (15%); 22 (11.3) | 1 (10%); 10 (0) | 4 (13%); 21 (10.5) |
| No, n (%) | 8 (89%) | 10 (77%) | 9 (90%) | 27 (84%) |
| Missing, n (%) | 0 (0%) | 1 (8%) | 0 (0%) | 1 (3%) |

| **AT 3 MONTHS**  **Have parents/family taken time off work in last 3 months due to child's health?** | **Arm A** | **Arm B1** | **Arm B2** | **Total** |
| --- | --- | --- | --- | --- |
| Have you taken time off? |  |  |  |  |
| Yes, n (%); mean (SD) no. days | 0 (0%); 0 (0) | 0 (0%); 0 (0) | 0 (0%); 0 (0) | 0 (0%); 0 (0) |
| No, n (%) | 7 (100%) | 11 (92%) | 10 (100%) | 28 (97%) |
| Missing, n (%) | 0 (0%) | 1 (8%) | 0 (0%) | 1 (3%) |
|  |  |  |  |  |
| Has your partner taken time off? |  |  |  |  |
| Yes, n (%); mean (SD) no. days | 1 (14%); 1 (0) | 4 (33%); 27.8 (35.7) | 2 (20%); 33.5 (37.5) | 7 (24%); 25.6 (31.5) |
| No, n (%) | 6 (86%) | 7 (58%) | 8 (80%) | 21 (72%) |
| Missing, n (%) | 0 (0%) | 1 (8%) | 0 (0%) | 1 (3%) |
|  |  |  |  |  |
| Have other family members taken time off? |  |  |  |  |
| Yes, n (%); mean (SD) no. days | 0 (0%); 0 (0) | 0 (0%); 0 (0) | 1 (10%); 10 (0) | 1 (3%); 10 (0) |
| No, n (%) | 7 (100%) | 11 (92%) | 9 (90%) | 27 (93%) |
| Missing, n (%) | 0 (0%) | 1 (8%) | 0 (0%) | 1 (3%) |

| **AT 6 MONTHS**  **Have parents/family taken time off work in last 3 months due to child's health?** | **Arm A** | **Arm B1** | **Arm B2** | **Total** |
| --- | --- | --- | --- | --- |
| Have you taken time off? |  |  |  |  |
| Yes, n (%); mean (SD) no. days | 0 (0%); 0 (0) | 0 (0%); 0 (0) | 0 (0%); 0 (0) | 0 (0%); 0 (0) |
| No, n (%) | 7 (100%) | 10 (91%) | 9 (100%) | 26 (96%) |
| Missing, n (%) | 0 (0%) | 1 (9%) | 0 (0%) | 1 (4%) |
|  |  |  |  |  |
| Has your partner taken time off? |  |  |  |  |
| Yes, n (%); mean (SD) no. days | 0 (0%); 0 (0) | 2 (18%); 30 (0) | 0 (0%); 0 (0) | 2 (7%); 30 (0) |
| No, n (%) | 7 (100%) | 8 (73%) | 9 (100%) | 24 (89%) |
| Missing, n (%) | 0 (0%) | 1 (9%) | 0 (0%) | 1 (4%) |
|  |  |  |  |  |
| Have other family members taken time off? |  |  |  |  |
| Yes, n (%); mean (SD) no. days | 0 (0%); 0 (0) | 0 (0%); 0 (0) | 0 (0%); 0 (0) | 0 (0%); 0 (0) |
| No, n (%) | 7 (100%) | 10 (91%) | 9 (100%) | 26 (96%) |
| Missing, n (%) | 0 (0%) | 1 (9%) | 0 (0%) | 1 (4%) |
