# Supplementary material for: Can provision of near vision glasses as an early intervention improve visual outcomes in infants at risk of perinatal brain insult? The Babies in Glasses (BiG) randomised feasibility trial
Source: BMJ Open. 2026 Feb 16;16(2):e107894. doi: 10.1136/bmjopen-2025-107894 (PMC12911751; doi:10.1136/bmjopen-2025-107894)
Supplement: Supplementary file 2 [file bmjopen-2025-107894.reviewer_comments.pdf]

PEER REVIEW HISTORY

BMJ Open publishes all reviews undertaken for accepted manuscripts. Reviewers are asked to complete a checklist review form and are provided with free text boxes to elaborate on their assessment. These free text comments are reproduced below.

ARTICLE DETAILS

Title (Provisional)

Can provision of near vision glasses as an early intervention improve visual outcomes in infants at risk of perinatal brain insult? The Babies in Glasses (BiG) randomized feasibility trial.

Authors

Bullaj, Raimonda; Dyet, Leigh; Mitra, Subhabrata; Bunce, Catey; Clarke, Caroline S; Saunders, Kathryn; Dale, Naomi; Horwood, Anna; Williams, Cathy; St Clair Tracy, Helen; Marlow, Neil; bowman, richard

---

VERSION 1 - REVIEW

**Reviewer**	**1**
**Name**	**Yap, Tiong Peng**
**Affiliation**	**IGARD Vision Therapy Center**
**Date**	**01-Nov-2025**
**COI**	**None**

This present study evaluated the feasibility of recruiting patients to examine the effect of near vision glasses in young infants at risk of cerebral visual impairment. The authors examined the recruitment and retention of the study participants, glasses compliance, and the testing protocol. They concluded that an RCT is feasible, and recommended some methodological modifications for a future larger study. The infants were prescribed with near glasses, but the prescribing philosophy and approaches are not fully explained in the manuscript. For example, it is not clear if hyperopia and astigmatic powers are fully prescribed. It is important for the authors to clarify this because there is a tendency for clinicians to under-prescribe glasses to infants in real world settings. It is interesting that the “standard treatment” was no glasses. Can the authors clarify why distance vision glasses was not considered? In the introduction section, the authors cited Hubel’s work on visual deprivation but this reference is not quite suitable in the context of vision correction (glasses) in humans.

**Reviewer**	**2**

**Name**	**Trevathan, Edwin**
**Affiliation**	**Vanderbilt University Medical Center**
**Date**	**17-Nov-2025**
**COI**	**None**

The authors have identified an important, often overlooked disability, cortical visual impairment, that may be reduced in severity by probably safe and low cost intervention. The rationale for the study is well-stated, and the authors have demonstrated the feasibility of a full clinical trial to determine the efficacy of their proposed intervention.

VERSION 1 - AUTHOR RESPONSE

Reviewer: 1
Dr. Tiong Peng Yap, IGARD Vision Therapy Center
Comments to the Author:

This present study evaluated the feasibility of recruiting patients to examine the effect of near vision glasses in young infants at risk of cerebral visual impairment. The authors examined the recruitment and retention of the study participants, glasses compliance, and the testing protocol. They concluded that an RCT is feasible, and recommended some methodological modifications for a future larger study. The infants were prescribed with near glasses, but the prescribing philosophy and approaches are not fully explained in the manuscript. For example, it is not clear if hyperopia and astigmatic powers are fully prescribed. It is important for the authors to clarify this because there is a tendency for clinicians to under-prescribe glasses to infants in real world settings. It is interesting that the "standard treatment" was no glasses. Can the authors clarify why distance vision glasses was not considered? In the introduction section, the authors cited Hubel's work on visual deprivation but this reference is not quite suitable in the context of vision correction (glasses) in humans.

Reviewer 1.
Thank you. Our prescribing philisophy is to compare 1) a prescription which gives the best focus possible for near vision (33cm) which is full cycloplegic refraction, including any cylinder, plus 3.00D near

add with 2) current standard of care (no glasses). Current standard of care is that babies are not routinely refracted and, even if they are, they are only precribed glasses for relatively high refractive error as indicated in clinical guidelines which is why for instance we did not give distance glasses to the control group. We have now explained this prescribing philosophy and the exact prescribing prototocol in the last paragraph of the introduction, page 4 and methods section, page 5 para 4.
We agree that the Hubel and Wiesel work referred to deprivation amblyopia but it was extended and applied to other types of amblyopia hence the practice of early occlusion for strabismic amblyopia or early surgery for infantile esotropia and to ametropic amblyopia which is why there are prescribing guidelines for glasses for infants with high refractive error. We have modified the second paragraph of the introduction page 4 to explain how the orginal work on deprivation amblyopia has been applied to different types of amblyopia including that caused by early onset refractive error.

VERSION 2 - REVIEW

**Reviewer**	**1**
**Name**	**Yap, Tiong Peng**
**Affiliation**	**IGARD Vision Therapy Center**
**Date**	**20-Dec-2025**
**COI**	

The authors have addressed reviewer's comments. There are just some minor comments: To maintain consistency, the authors should choose either "glasses" or "spectacles" and use the same term throughout the manuscript. Line 23 will need a reference to support the statement about uncorrected refractive errors. To improve this, the author should explain why optical correction is important from a neurophysiological viewpoint. For example, the authors can look at Yap, et al., 2020 (IOVS). Line 26: Add a comma after the word "However". Line 34-35: When using an abbreviation for the first time, the full term should be followed by the abbreviation in parentheses to ensure clarity for the reader.

VERSION 2 - AUTHOR RESPONSE

Reviewer: 1
Dr. Tiong Peng Yap, IGARD Vision Therapy Center
Comments to the Author:

The authors have addressed reviewer's comments. There are just some minor comments: To maintain consistency, the authors should choose either "glasses" or "spectacles" and use the same term throughout the manuscript. Line 23 will need a reference to support the statement about uncorrected refractive errors. To improve this, the author should explain why optical correction is important from a neurophysiological viewpoint. For example, the authors can look at Yap, et al., 2020 (IOVS). Line 26: Add a comma after the word "However". Line 34-35: When using an abbreviation for the first time, the full term should be followed by the abbreviation in parentheses to ensure clarity for the reader.

Thank you- we have removed the 2 instances of the word 'spectacles' and replaced with 'glasses'.
We have inserted the above reference to show how neurophysiological correlates of refractive amblyopia have been shown.
We have added the requested comma and spelled dioptres in full on first use.
